# Overexpression of Fas and FasL Is Associated with Infectious Complications and Severity of Experimental Severe Acute Pancreatitis by Promoting Apoptosis of Lymphocytes

**DOI:** 10.1007/s10753-014-9847-8

**Published:** 2014-02-25

**Authors:** Liao Pinhu, Yueqiu Qin, Bin Xiong, Yanwu You, Jun Li, Suren R. Sooranna

**Affiliations:** 1 Intensive Care Unit, Affiliated Hospital of Youjiang Medical University for Nationalities, No. 18 Zhongshan Road II, 533000 Baise, Guangxi Zhuang Autonomous Region China; 2 Intensive Care Unit, People’s Hospital of Guangxi Zhuang Autonomous Region, 531000 Nanning, China; 3 Department of Surgery and Cancer, Imperial College London, Chelsea and Westminster Hospital, London, SW10 9NH UK

**Keywords:** severe acute pancreatitis, CD95, immunosuppression, infection, apoptosis

## Abstract

This study investigated the relationship of Fas and Fas ligand (FasL) expression and apoptosis of lymphocytes in relation to the pathogenic immune response and infectious complications observed in experimental severe acute pancreatitis in mice. Forty male Balb/c mice were randomly divided into control, mild (MAP), and severe acute pancreatitis (SAP) groups. Overexpression of Fas/FasL messenger ribonucleic acid (mRNA) and protein was observed in spleen-derived lymphocytes in SAP (*p* < 0.01). Apoptosis of these resulted in a depletion of circulating lymphocytes in this group (*p* < 0.05). A further significant change in the SAP group with infectious complications was observed. A positive relationship was found between the Fas/FasL expression and lymphocyte apoptosis, and negative relationships were observed between Fas/FasL expression and CD4^+^ and CD19^+^ lymphocytes and the CD4^+^/CD8^+^ ratio in SAP mice (*p* < 0.01). The results suggest that the overexpression of Fas/FasL is associated with infectious complications and severity of experimental severe acute pancreatitis by promoting apoptosis of lymphocytes.

## INTRODUCTION

Severe acute pancreatitis (SAP) is a disease that develops from local pancreatic inflammation to overwhelming systemic inflammation. It is associated with severe infectious complications and multiple organ failure. Necrotic pancreatic tissue in SAP is one of the main reasons that lead to systemic inflammation and mortality [1, 2]. It has been widely accepted that uncontrolled inflammatory response plays a key role in the occurrence of infection and sepsis [3, 4] and that the immune response such as immunosuppression is also involved [5, 6]. With the development of systemic inflammation, pro- and anti-inflammatory cytokines are released into the circulation, causing compensatory anti-inflammatory response syndrome (CARS) and subsequent immune deficiency or immunosuppression [7–9] which renders the host susceptible to secondary infections and to systemic sepsis [10]. However, the molecular mechanisms involved in the pathogenesis of this disease remain poorly understood. Extensive apoptosis of lymphocytes and intestinal epithelial cells in patients with sepsis, shock, and multiple organ dysfunction has been found, and these results suggested that the changes of immune status contribute to the immunosuppression [11]. Prevention of lymphocyte apoptosis has been shown to improve immunological impairment and survival in septic mice [12, 13].

The Fas molecule (also known as CD95 or Apo-1) is a type I transmembrane protein, and Fas ligand (FasL) is the natural ligand of Fas and is a type II transmembrane protein. Fas is widely expressed, while FasL expression is observed mainly in activated lymphocytes and vascular endothelial cells [14]. A previous work has demonstrated that Fas and FasL play critical roles in delivering death signals to the immune system, and interactions of Fas–FasL can initiate the death signal pathway leading to lymphocyte apoptosis [15–17]. Mutation or downregulation of the expression of Fas and FasL genes resulted in lymphocyte proliferation and autoimmune disease [18, 19]. In contrast, upregulation of the expression of Fas and FasL may cause excessive apoptosis of lymphocytes leading to immunological impairment and immunosuppression [20]. Recent studies have shown that Fas/FasL-mediated apoptosis of pancreatic acinar cells, intestinal epithelial cells, and Kupffer cells occur in acute pancreatitis [21–23]. Decreased numbers of peripheral blood and spleen lymphocytes had been found in SAP rats [24]. The numbers of CD4^+^, CD8^+^, and CD20^+^ T lymphocytes in SAP patients were significantly lower than the normal ranges. The numbers of CD4^+^ and CD8^+^ T lymphocytes decreased more significantly in patients with infection [5]. Although Fas and FasL have been shown as the main factors that lead to apoptosis of lymphocytes, the expression levels of Fas/FasL and apoptosis in spleen lymphocytes of SAP need to be studied further. The relationships between Fas/FasL expression, apoptosis of spleen lymphocytes, changes of immune status, and infectious complications in experimental SAP remain largely unknown.

The aim of this study was to investigate the role of Fas/FasL expression and the apoptosis of lymphocytes, the changes of immune status, and the infectious complications in an experimental severe acute pancreatitis mice model.

## MATERIALS AND METHODS

### Animals

The study was approved by the Committee on Animal Care and Use of Youjiang Medical University for Nationalities and was performed according to the National Institute of Health guidelines. Forty male Balb/c mice (8–12 weeks old) weighing 25 to 30 g were used and were maintained at 2,225 °C and 40–60 % relative humidity using a 12-h light/dark cycle. They were fasted for 12 h prior to experiments but were allowed free access to tap water.

### Experimental Protocol

The animals were randomly divided into a control group (injected with normal saline, *n* = 10) and two groups whereby pancreatitis was induced with caerulein. The mild acute pancreatitis (MAP) group (*n* = 10) was induced by six hourly intravenous injections of caerulein (5 μg/kg; C9026 Sigma-Aldrich, USA) [25, 26]. The SAP group (*n* = 20) was divided into two subgroups according to the result of bacterial culture: a subgroup with infection complications (*n* = 12) and a subgroup without infectious complication (*n* = 8). The mice in the SAP group received six hourly intraperitoneal injections of caerulein (50 μg/kg) and then a single intraperitoneal injection of 10 mg/kg lipopolysaccharide (LPS; *Escherichia coli* 055:B5, L2880, Sigma-Aldrich, USA) after the last caerulein injection as previously described [27–30]. Animals were anesthetized with 50 mg/kg pentobarbital 12 h after the last injection. Blood samples were collected by cardiac puncture for measuring serum amylase activity and lipase activity and to detect circulating lymphocyte subsets. The spleen was harvested; a part of which was fixed and embedded in paraffin wax for immunohistochemical and TdT-mediated dUTP nick-end labeling (TUNEL) staining, and the other part was immediately processed for splenic lymphocyte suspensions as described below. The pancreas was also carefully removed for histological evaluations and bacterial culture. The mesenteric lymph node (MLN) complex was harvested for bacterial culture. All of these procedures were performed under aseptic conditions.

### Biochemical Assays

Blood samples (400 μL) were collected by cardiac puncture and transferred into 0.5-mL centrifugation tubes, allowed to clot, and then centrifuged at 3,000×*g* for 5 min. The serum was collected and stored. Serum amylase and lipase activities were determined using commercially available kits (Sigma-Aldrich, USA).

### Histological Examination

The pancreas was fixed in 10 % neutral formaldehyde, embedded in paraffin wax, and then was sectioned (4 μm thickness) and stained with hematoxylin and eosin (HE). The sections were examined and scored by two pathologists who were blinded to the experimental protocol. A scoring system previously described by Schmidt *et al.* [31] was used to score the tissues for pancreatic edema, acinar necrosis, inflammation, and hemorrhage.

### Bacterial Examination

The MLN complex and the other parts of the pancreas were weighed and transferred into sterile tubes containing 0.5 mL of precooled phosphate-buffered saline (PBS) and were then homogenized with a glass grinder. The homogenates were placed into brain–heart culture medium and plated onto sheep blood agar, MacConkey agar, and Columbia calistin nalidixic acid (CNA) agar (Becton Dickinson and Company, USA). After 48 h of incubation at 37 °C, colonies were identified and results were expressed as colony-forming units (CFUs) per gram of tissue.

### Preparation of Lymphocytes Derived from the Spleen

Single cell suspensions of spleen were made by grinding the spleen on nylon nets (200 mesh) in 35-mm petri dishes containing 5 mL of mouse lymphocyte isolation liquid (Dakewe, Shenzhen, China). The cell suspensions of spleen were transferred into centrifuge tubes and covered with 300 μL of RPMI 1640 culture medium. Lymphocytes were harvested using density gradient centrifugation (at 800×*g* for 20 min). After counting and observing cell morphology under a microscope, RNA and protein of these lymphocytes were immediately extracted as follows.

### Real-Time Polymerase Chain Reaction (PCR)

Total RNA of splenic lymphocytes was extracted with chloroform and TRIzol (Invitrogen, USA) according to the TRIzol kit protocol. RNA (2 μg) was reverse transcribed (RT) into complementary deoxyribonucleic acid (cDNA) using M-MLV reverse transcriptase with Oligo dT (Invitrogen, USA). cDNA was aliquoted and stored at −80 °C until used. Mouse Fas, FasL, and actin, beta (ACTB) primers were designed using Primer Express software (version 3.0). Polyacrylamide gel electrophoresis (PAGE) level purification primers of mouse Fas (NM_007987.2), FasL (NM_010177.4), and ACTB (NM_007393.3) were synthesized (Invitrogen Company, Shanghai, China). Fas primer sequence is forward 5′-ATGCACACTCTGCGATGAAG-3′ and reverse 5′-CAGTGTTCACAGCCAGGAGA-3′; FasL primer sequence is forward 5′-GCAGAAGGAACTGGCAGAAC-3′ and reverse 5′-TTAAATGGGCCACACTCCTC-3′; and ACTB primer sequence is forward 5′-GGGAATGGGTCAGAAGGACT-3′ and reverse 5′-CTTCTCCATGTCGTCCCAGT-3′. The expression levels of Fas and FasL were semiquantitatively measured by real-time PCR (Bio-Rad iQ5, USA) using QuantiFast SYBR green PCR kit (cat. 204054, Qiagen, Germany). After 5 min of initial activation at 95 °C, PCR was carried out for 40 cycles at 95 °C for 10 s and 61.3 °C for 30 s. ACTB was performed simultaneously and used as the housekeeping gene. The threshold cycle (Ct) value was measured, and the comparative gene expression was calculated by 2^−ΔΔCt^ method as described previously [32]. Two percent agarose gel electrophoresis was used to identify amplification products.

### Western Blot Analysis

Splenic lymphocytes were diluted in lysis buffer (50 mM Tris–HCl, pH 7.4, 150 mM NaCl, 1 % Triton X-100, 0.1 % sodium dodecyl sulfate (SDS), 2 mM ethylenediaminetetraacetic acid (EDTA), 0.1 mM EGTA, 5 mM NaF, 1 mM Na_3_VO_4_, 5 mM Na_2_PO_4_, and 1× proteinase inhibitor cocktail (Beyotime Institute of Biotechnology, China)) on ice for 30 min and then centrifuged (12,000 rpm, 20 min) at 4 °C. The supernatants were collected, aliquoted, and stored at −80 °C until used. Western blot analysis of Fas and FasL was conducted and quantified as described [33, 34]. After separating on SDS-PAGE gel electrophoresis, proteins were transferred to polyvinylidene difluoride (PVDF) membranes (Merck Millipore, USA) and blocked with 5 % nonfat milk for 2 h at room temperature. The following antibodies were used as primary antibodies: rabbit anti-Fas antibody (ab82419, Abcam Ltd, Hong Kong, 1:200 dilution), rabbit anti-FasL antibody (sc-834, Santa Cruz Biotechnology, USA, 1:200 dilution), and mouse anti-beta actin monoclonal antibody (AA128, Beyotime Institute of Biotechnology, China, 1:1,000 dilution), followed by the goat polyclonal secondary antibody to rabbit IgG-H&L-HRP (ab6721, Abcam Ltd, Hong Kong, 1:1,000 dilution) or to mouse IgG-H + L-HRP (A0216, Beyotime Institute of Biotechnology, China, 1:1,000 dilution). Blots were exposed for 60 s to BeyoECL Plus (P0018, Beyotime Institute of Biotechnology, China). The images of Western blots were scanned by ChemiDoc^TM^ XRS (Bio-Rad, USA), acquired, and analyzed by using Quantity One software (Bio-Rad, USA). Protein levels were expressed as density and were normalized to beta-actin for statistical comparisons [33].

### Immunohistochemistry

The spleen was fixed in 10 % neutral formaldehyde and embedded in paraffin wax. The 4-μm-thick sections were dewaxed in xylene and rehydrated by taking through a graded series of ethanol. The endogenous peroxidase activity was blocked with 3 % hydrogen peroxide for 15 min. Sections were incubated with rabbit anti-Fas antibody (ab82419, Abcam Ltd, Hong Kong, 1:100 dilution) or rabbit anti-FasL antibody (sc-834, Santa Cruz Biotechnology, USA, 1:200 dilution) overnight at 4°C and then incubated with goat polyclonal secondary antibody to rabbit IgG-H&L-HRP (ab6721, Abcam Ltd, Hong Kong, 1:500 dilution) for 30 min at 37 °C. Negative controls were incubated with normal rabbit serum instead of the first antibody. Finally, sections were stained by using a 3,3'-diaminobenzidine (DAB) kit (Maixin Biotechnology Company, China) and counterstained with hematoxylin, dehydrated through a graded series of ethanol solutions, cleared with xylene, and mounted with coverslips. The analysis was performed using a scoring system as described previously [35]. All the slides were scored by two pathologists who were blinded to the pathology and experimental protocol.

### Apoptosis TUNEL Assay

The TUNEL assay was performed using an *in situ* cell death detection kit, peroxidase-based secondary detection systems (POD) (11684817910, Roche Applied Science, Germany), according to the manufacturer’s instructions. After incubation with the TUNEL reaction mixture containing TdT and fluorescein-dUTP, the sections of the spleen were analyzed under a fluorescence microscope (Olympus, Japan, excitation wavelength in the range of 450–500 nm and detection in the range of 515–565 nm). The cells were finally incubated with converter-POD, stained by using a DAB kit (Maixin Biotechnology Company, China), and counterstained with hematoxylin. A light microscope was used to count cells in ten randomly selected high-power field areas (×400) without necrosis per slide. The apoptosis index (AI) was defined as the percentage of TUNEL-positive cells to all counted cells.

### Flow Cytometry Analysis

Blood samples were collected into sterile tubes containing EDTA anticoagulant. Fifty microliters of blood was incubated (in the absence of light) in TruCount^TM^ tubes with lymphocyte subset antibodies (anti-mouse CD3 APC-eFluor 780 17A2, anti-mouse CD4 APC GK1.5, anti-mouse CD8a PE 53-6.7, and anti-mouse CD19 PE-Cy7 eBio1D3 (1D3) were from eBioscience, USA ) at 25 °C for 15 min. Matched labeled isotype antibodies were used as negative controls (rat IgG2b K Isotype control APC-eFluor 780, rat IgG2b K Isotype control APC, rat IgG2a K Isotype control PE, and rat IgG2a K Isotype control PE-Cy7 were from eBioscience, USA). Then, the samples were treated with 450 μL 1× BD FACS lysing solution (BD Company, USA). After the erythrocytes were lysed, 10,000 cells along with beads were acquired on a FACSCanto™ II flow cytometer (BD Company, USA). The results were analyzed using FACSDiva software (BD Company, USA).

### Statistical Analysis

Results are expressed as mean ± SEM. After the homogeneity test of variance (Levene’s test), the results were analyzed by one-way ANOVA followed by *post hoc* multiple comparisons (Bonferroni test); *p* < 0.05 was accepted as statistically significant. Correlation between the expression levels of Fas and FasL protein and lymphocyte apoptosis and CD4^+^ and CD19^+^ lymphocytes and CD4^+^/CD8^+^ ratio was evaluated by Pearson’s correlation coefficient. *P* < 0.05 was considered statistically significant.

## RESULTS

### Biochemical and Histological Findings

**Fig. 1:**
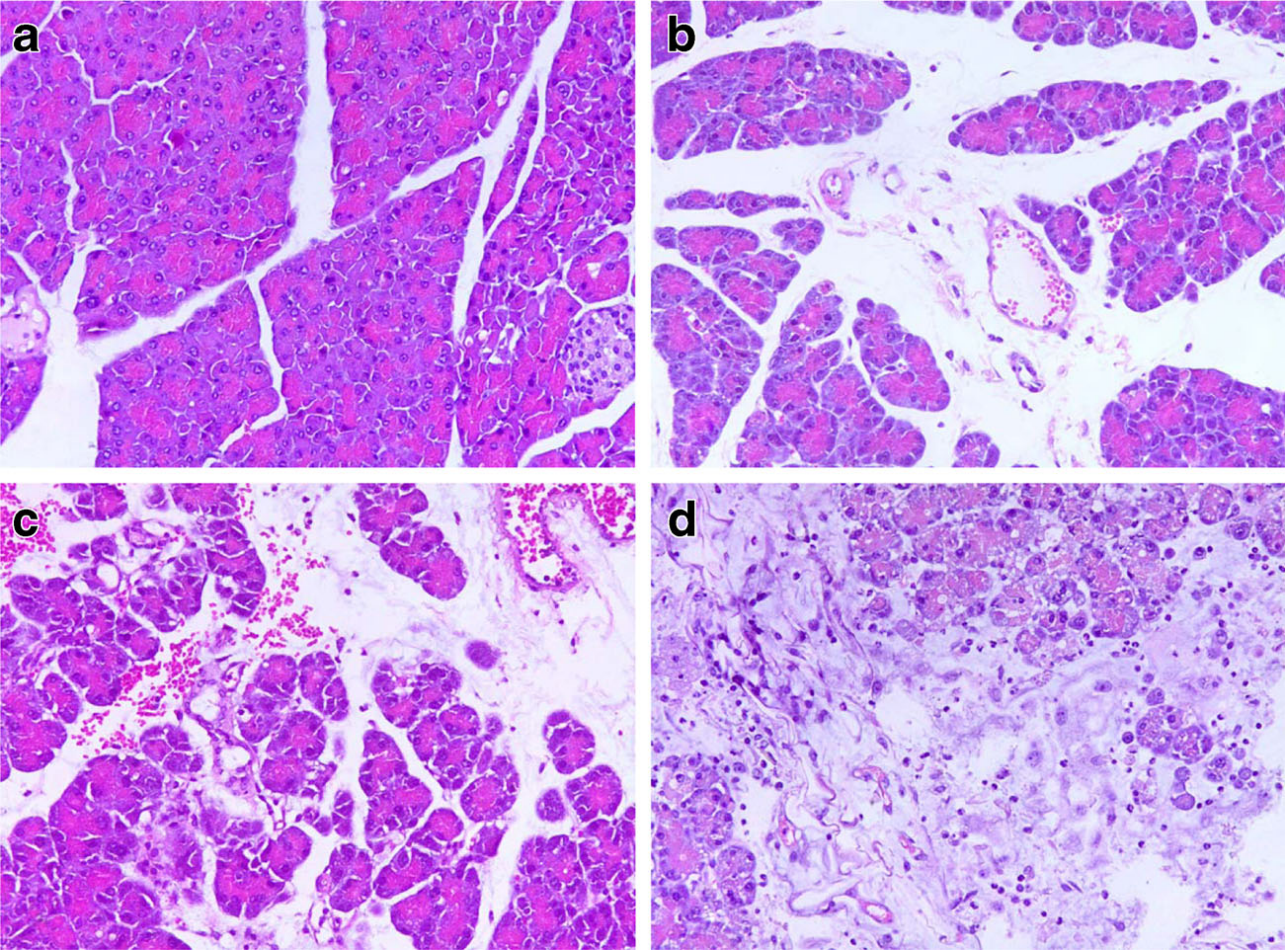
Hematoxylin and eosin staining of pancreas tissue sections. **a** Animals of the control group displayed normal pancreatic histology. **b** Animals of the caerulein (Cn) group (MAP group) treated with caerulein (6 × 5 μg/kg) showed mild edematous pancreatitis characterized by interstitial edema and slight infiltration of inflammatory cells but without obvious acinar necrosis and parenchymal hemorrhage. **c**, **d** Animals of the Cn + LPS group (SAP group) treated with caerulein (6 × 50 μg/kg) and lipopolysaccharide (1 × 10 mg/kg) showed the features of severe acute pancreatitis characterized by severe interstitial edema of interlobular and intralobular spaces, extensive infiltration with inflammatory cells, obvious acinar necrosis, and parenchyma hemorrhage (original magnification ×400).

Intraperitoneal injection of caerulein and LPS was associated with significant increases in the serum levels of lipase and amylase (Table 1) and resulted in SAP. Histological examination of pancreas at 12 h after caerulein and LPS administration revealed tissue damage characterized by edema, acinar necrosis, inflammation, and hemorrhage (Table 2 and Fig. 1).

**Table 1:** Measurements of Serum of Lipase and Amylase Activities

	Control	Cn (MAP group)	Cn + LPS (SAP group)
Amylase (U/L)	242.90 ± 15.54	2,638.90 ± 166.84^a^	6,743.95 ± 283.67^ab^
Lipase (U/L)	61.20 ± 3.38	385.30 ± 23.960^a^	1,360.80 ± 57.14^ab^

*n* = 10 in control and Cn groups; *n* = 20 in Cn + LPS group

*Cn* caerulein (6 × 5 μg/kg); *Cn + LPS* caerulein (6 × 50 μg/kg) + lipopolysaccharide (1 × 10 mg/kg)

^a^ *p* < 0.01 *vs.* control group

^b^ *p* < 0.01 *vs.* Cn group

**Table 2:** Histological Scoring of Pancreatic Injury

	Edema	Acinar necrosis	Inflammation	Hemorrhage
Control	0.0	0.0	0.0	0.0
Cn (MAP group)	1.50 ± 0.13	0.10 ± 0.07	1.05 ± 0.12	0.0
Cn + LPS (SAP group)	3.05 ± 0.11^a^	2.68 ± 0.13^a^	2.83 ± 0.13^a^	1.28 ± 0.14^a^

*n* = 10 in control and Cn groups; *n* = 20 in Cn + LPS group

*Cn* caerulein (6 × 5 μg/kg); *Cn + LPS* caerulein (6 × 50 μg/kg) and lipopolysaccharide (1 × 10 mg/kg)

^a^ *p* < 0.01 *vs.* Cn group

### Infectious Complications in SAP

Bacterial cultures showed that no bacterium was observed from the MLN complex or the pancreas in both the control and MAP groups. In the SAP group, positive rates of bacterial culture from the MLN complex and pancreas were 45 % (9/20) and 60 % (12/20), respectively, at 12 h after caerulein and LPS administration. *E. coli* was cultured most frequently from the MLN (77 %, 7/9) and pancreas (83 %, 10/12), followed by *Proteus* characterized at 33 % (3/9) from MLN and 42 % (5/12) from pancreas. Positive rates of *Enterococcus* from MLN and pancreas were 45 % (4/9) and 33 % (4/12), respectively. Pancreatic infection was regarded as a sign of infectious complications in this study.

### Expression of Fas and FasL mRNA in Lymphocytes from the Spleen

**Fig. 2:**
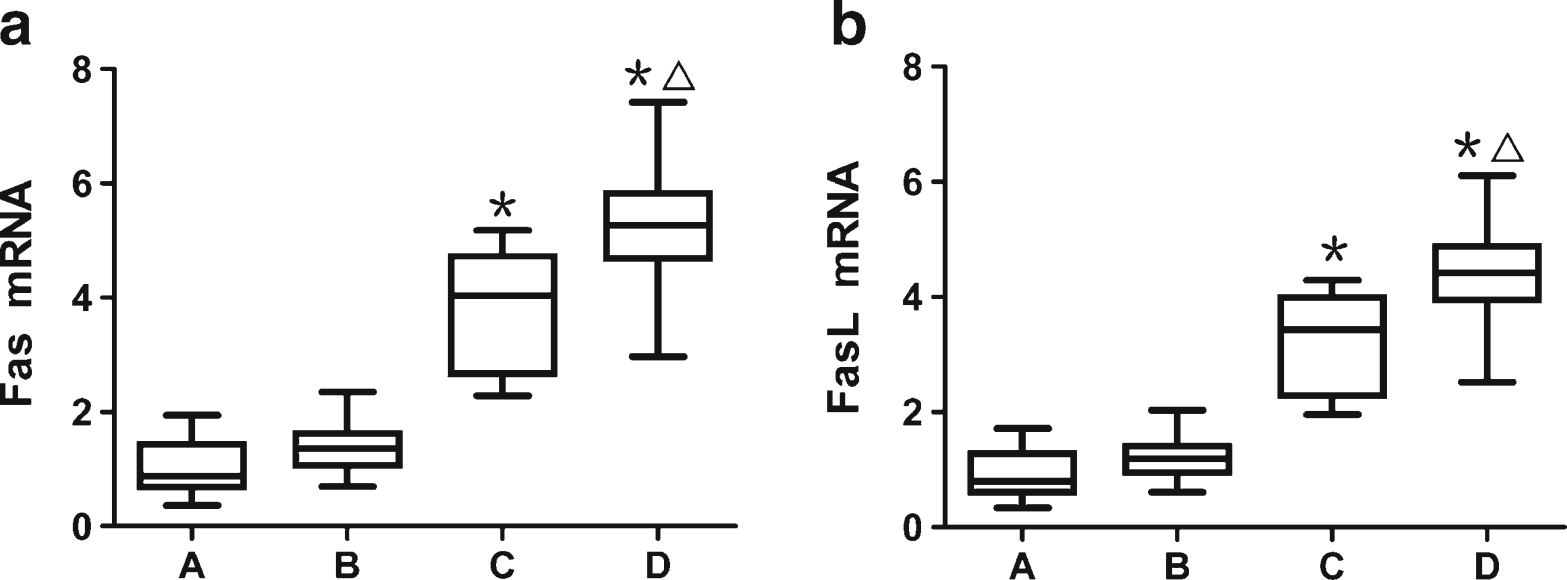
The semiquantitative analysis of **a** Fas mRNA and **b** FasL mRNA in splenic lymphocytes by real-time PCR. The relative gene expression was calculated by 2^−ΔΔCt^ method. ^*^ *p* < 0.01 *vs.* control group and MAP group; ^△^ *p* < 0.01 *vs.* SAP group without infectious complications (*A* control group; *B* MAP group; *C* SAP group without infectious complications; *D* SAP group with infectious complications).

Analysis by real-time PCR indicated that the expression levels of Fas and FasL messenger ribonucleic acid (mRNA) were increased significantly in the SAP group compared with the MAP and control groups (*p* < 0.01 in all cases). There was a further increase in the SAP group with infectious complications compared to those without (*p* < 0.01 in all cases). The expression of Fas and FasL mRNA also increased in the MAP group, but there was no significant difference compared with the control group (Fig. 2a, b).

### Localization and Expression Level of Fas and FasL Protein in Lymphocytes from the Spleen

**Fig. 3:**
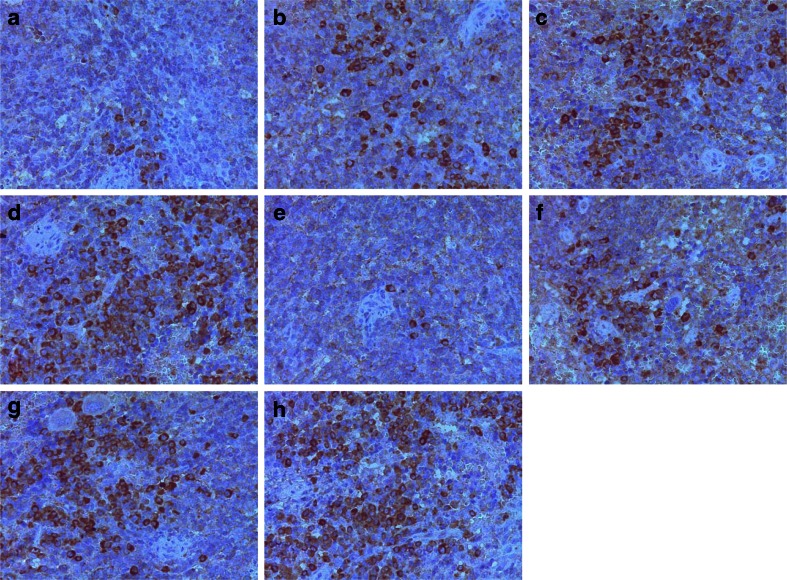
Immunohistochemistry of Fas and FasL protein of spleen tissue sections. Immunohistochemical analysis shows that Fas protein (**a**–**d**) and FasL protein (**e**–**h**) accumulate in the cell membrane and cytoplasm of splenic lymphocytes. Compared with the control (**a**, **e**) and MAP groups (**b**, **f**), the staining intensity and number of cells expressing Fas and FasL increased markedly in the SAP group (**c**, **d**, **g**, **h**), especially in those animals with infectious complications (**d**, **h**; original magnification ×400).

**Fig. 4:**
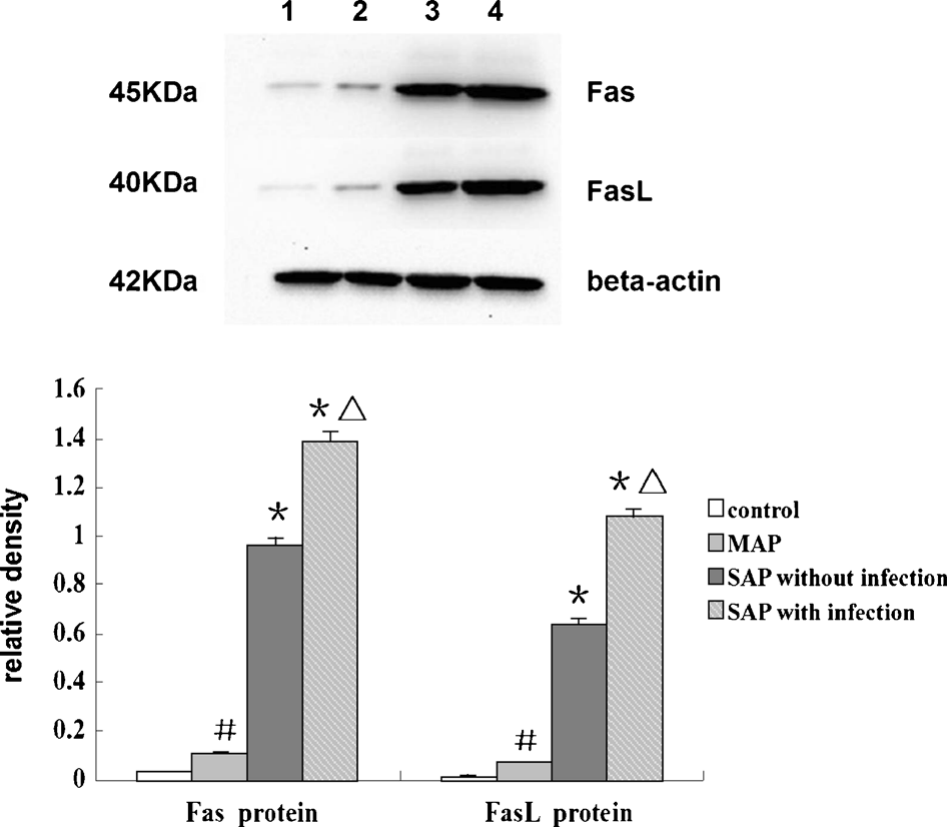
Western blot analysis of Fas and FasL protein in splenic lymphocytes. Protein levels were expressed as density and were normalized to beta-actin. ^*^ *p* < 0.01 *vs.* control and MAP groups; ^△^ *p* < 0.01 *vs.* SAP group without infectious complications; ^**#**^ *p* < 0.01 *vs.* control group (*1* control group; *2* MAP group; *3* SAP group without infectious complications; *4* SAP group with infectious complications).

Immunohistochemistry analysis revealed that Fas and FasL proteins were expressed in the cell membrane and cytoplasm of splenic lymphocytes. The staining intensity and number of cells expressing Fas and FasL increased markedly in the SAP group, especially in those mice with infectious complications, when compared to the control group (Fig. 3). The expression levels of Fas and FasL protein in splenic lymphocytes were significantly increased in the SAP group as detected by Western blotting, and these changes were more significant in those mice with infectious complications (*p* < 0.01 in all cases; Fig. 4).

### Apoptosis in Lymphocytes from the Spleen

**Fig. 5:**
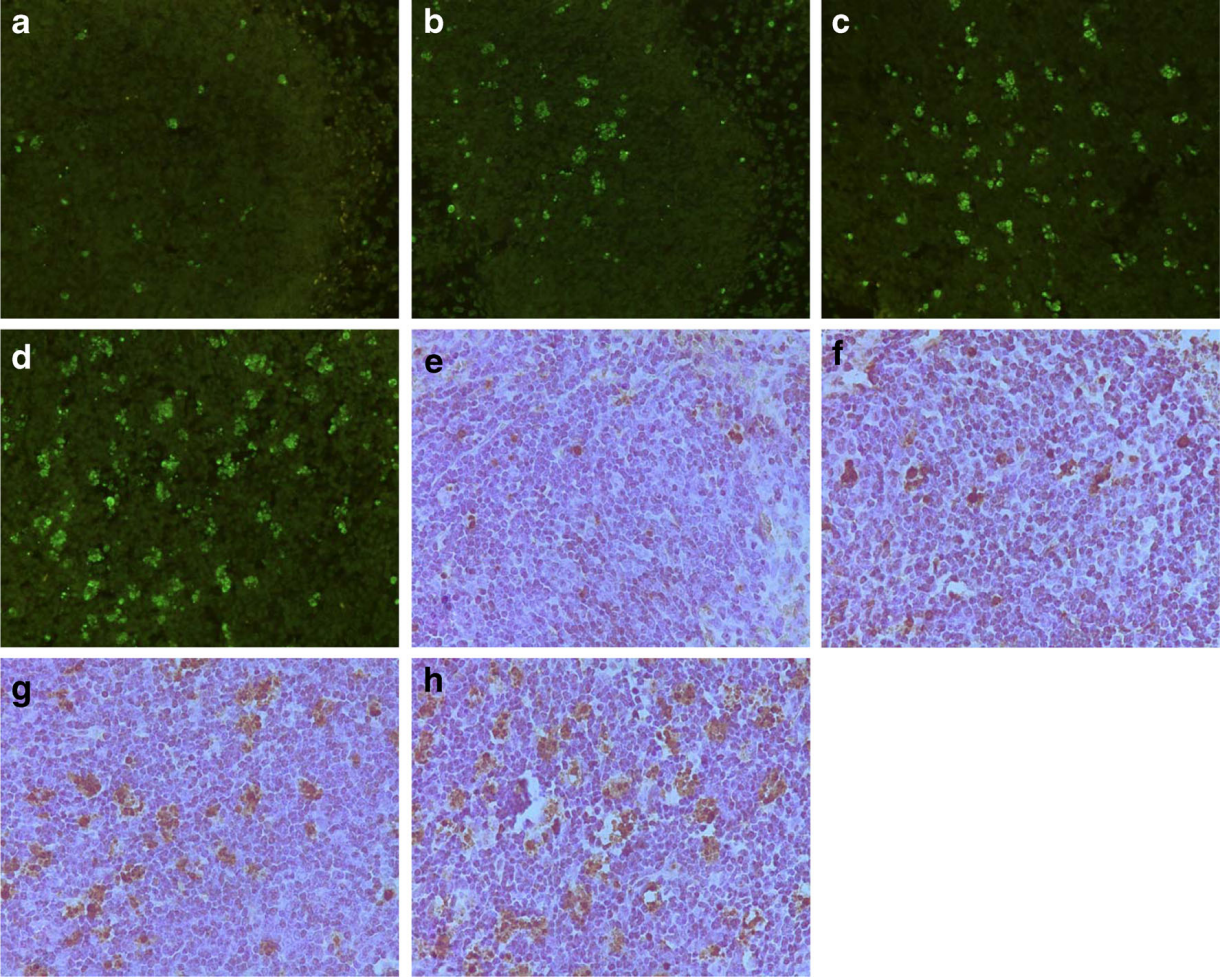
TUNEL staining of splenic lymphocytes of spleen tissue sections. **a**–**d** TUNEL-positive splenic lymphocytes displayed brilliant green fluorescence under a fluorescence microscope. **e**–**h** After sections were incubated with the converter-POD and counterstained with hematoxylin, apoptotic nuclei were stained brown when observed under a light microscope. Only a few apoptotic cells were visible in the control (**a**, **e**) and MAP groups (**b**, **f**). The number of apoptotic cells increased markedly in the SAP group (**c**, **d**, **g**, **h**), especially in those animals with infectious complications (**d**, **h**; original magnification ×400).

**Fig. 6:**
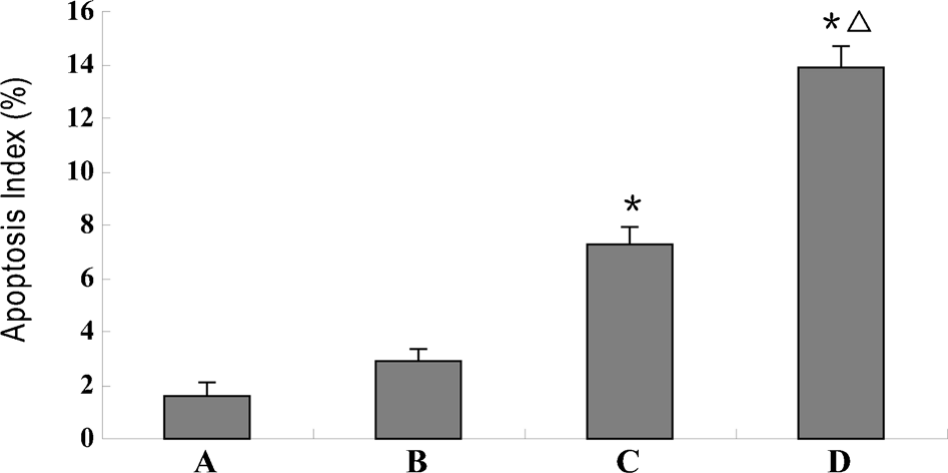
The apoptosis index (AI) of splenic lymphocytes. Apoptosis was assessed by TUNEL. ^*^ *p* < 0.01 *vs.* control and MAP groups; ^△^ *p* < 0.01 *vs.* SAP group without infectious complications (*A* control group; *B* MAP group; *C* SAP group without infectious complications; *D* SAP group with infectious complications).

TUNEL-positive splenic lymphocytes displayed brilliant green fluorescence under the fluorescence microscope (Fig. 5a–d). After incubation with the converter-POD and counterstained with hematoxylin, apoptotic nuclei were stained brown when observed under a light microscope (Fig. 5e–h). The number of positive cells increased markedly in the SAP group, especially in those mice with infectious complications, when compared to the control group. The AIs of the SAP group were significantly greater than those of the control and MAP groups (*p* < 0.01). There was a further increase in AI in the SAP group with infectious complications when compared to those without (*p* < 0.01; Fig. 6).

### Changes in Circulating Lymphocyte Subsets

**Fig. 7:**
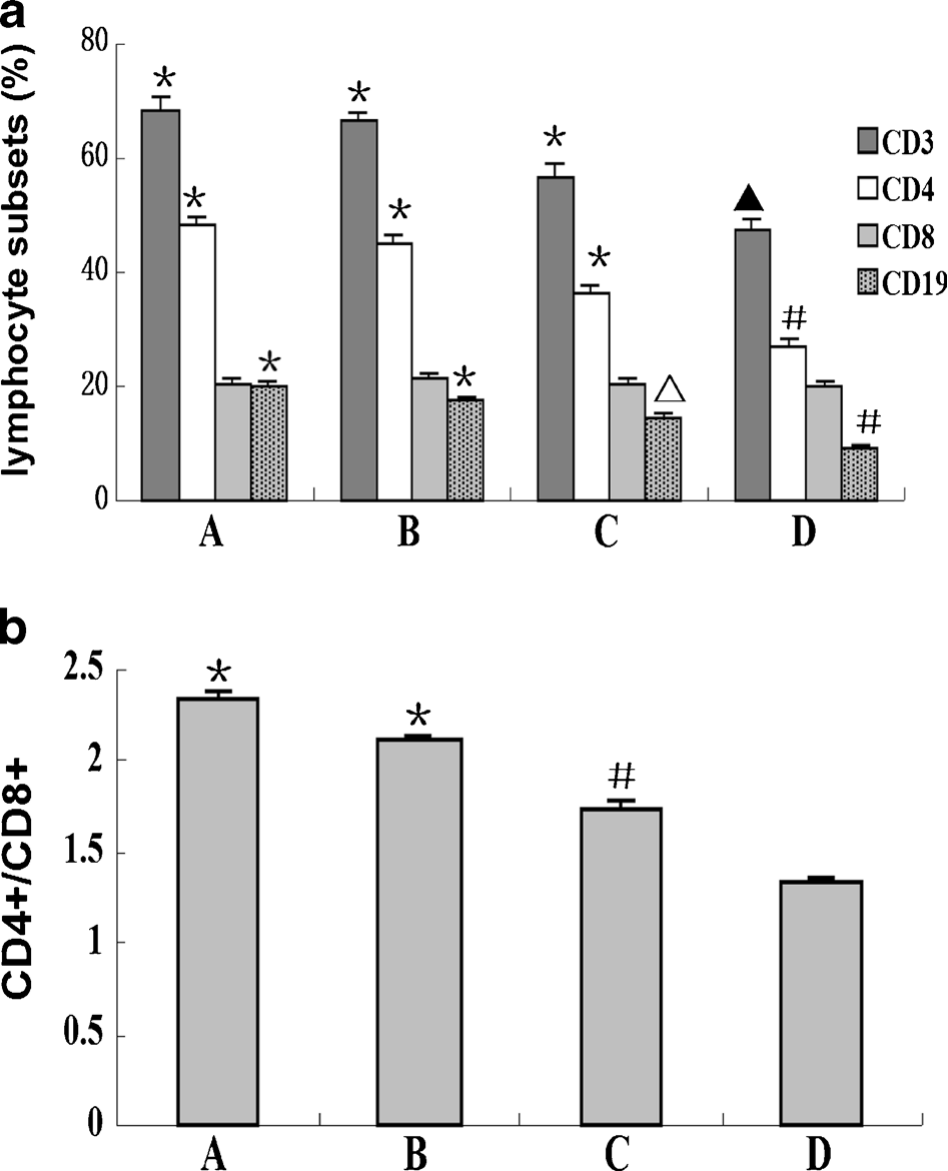
Changes of circulating **a** lymphocyte subsets and **b** CD4^+^/CD8^+^ ratio. ^*^ *p* < 0.01 *vs.* SAP group with or without infectious complications and SAP group with infectious complications; ^△^ *p* < 0.05 *vs.* SAP group without infectious complications; ^△^ *p* < 0.01 *vs.* SAP group with infectious complications; ^**#**^ *p* < 0.01 *vs.* SAP group with infectious complications; *p* ^**▲**^ < 0.05 *vs.* SAP group with infectious complications (*A* control group; *B* MAP group; *C* SAP group without infectious complications; *D* SAP group with infectious complications).

The percentage of lymphocyte subsets (CD3^+^, CD4^+^, and CD19^+^) and the CD4^+^/CD8^+^ ratio were decreased significantly in the SAP group compared with the MAP and control groups (*p* < 0.01 or *p* < 0.05), and there was a further decrease in the SAP group with infectious complications when compared to those without (*p* < 0.01 or *p* < 0.05). No significant changes of lymphocyte subsets were found between the MAP and control groups, and there was no significant change in the percentage of CD8^+^ T-cell ratio between each group (Fig. 7a, b).

### Correlation Between the Expression of Fas and FasL Protein and Apoptosis of Lymphocytes and Lymphocyte Subsets

**Fig. 8:**
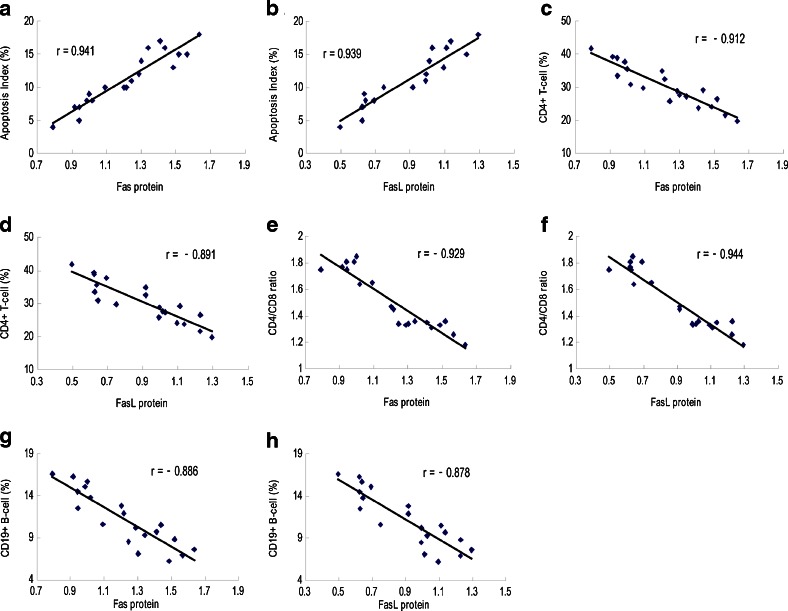
Correlations between the expression of Fas and FasL protein and apoptosis index (AI) and CD4^+^ and CD19^+^ lymphocytes and CD4^+^/CD8^+^ ratio. The data show a significant positive relationship between Fas and FasL protein expression and AI (**a**, **b**) and significant negative relationships between Fas and FasL protein expression and CD4^+^ T cell (**c**, **d**), CD4^+^/CD8^+^ ratio (**e**, **f**), and CD19^+^ B cell (**g**, **h**) in the SAP group (*p* < 0.05 in all cases).

There was a significant positive relationship between Fas/FasL protein expression and AI (Fig. 8a, b), and there was a significant negative relationship between Fas/FasL protein expression and CD4^+^ (Fig. 8c, d) and CD19^+^ (Fig. 8g, h) lymphocyte subsets as well as the CD4^+^/CD8^+^ ratio (Fig. 8e, f) in SAP group (*p* < 0.05 in all cases).

## DISCUSSION

The histological changes including edema, acinar necrosis, inflammation, and hemorrhage after the administration of caerulein and LPS in the SAP group in this study indicate the successful replication of the mouse model described in previous studies [27–30]. LPS clearly damages the functional intestinal barrier, which results in an increase of mucosal permeability, and this is considered to be a major promoter of bacterial translocation (BT) [36]. Intraperitoneal injection of LPS could mimic infection by increasing gut paracellular permeability and inducing BT [37–39]. Gut paracellular permeability was significantly higher at 6 h after LPS injection [39].

Lymphocytes play an important immunological role in acute pancreatitis. Significant reduction of lymphocytes may lead to immunological impairment. Several studies have reported depletion of circulating lymphocytes in acute pancreatitis [40–42], especially CD4-positive lymphocytes [43]. Significant decreases in CD4- and CD8-positive peripheral lymphocytes may cause immune suppression in the early phase of SAP and could be closely related to infectious complications [5]. In this study, ratios of circulating lymphocyte subsets were decreased significantly in the SAP group, including CD3^+^ cells, CD4^+^ T-cells, CD4^+^/CD8^+^ ratio, and CD19^+^ cells. There was a further decrease in the SAP group with infectious complications when compared to mice without infectious complication. While there was no significant change of lymphocyte subsets between the MAP and control groups and there was a small change in the CD8^+^ T-cells between each group, this result was consistent with an immunological impairment in the SAP group, especially in those with infectious complications.

Experiments have shown that the reduction of peripheral lymphocytes was primarily due to lymphocyte apoptosis [44]. Extensive apoptosis of lymphocytes, including B and T lymphocytes in peripheral blood and lymphocytes from the spleen and thymus, was observed during sepsis and SAP [12, 45, 46]. Prevention of lymphocyte apoptosis improves immunological impairment and survival in sepsis [12, 13]. Our study also found a significantly increased number of apoptotic lymphocytes and a high AI in the spleen of the SAP group, especially in those mice with infectious complications. Extensive apoptosis of lymphocytes may be the main mechanism for the reduction of lymphocyte subsets in SAP.

Apoptosis is important to maintain adequate lymphocyte development and homeostasis. It is well-known that differentiation, maturation, activation, and deletion of T and B cells in the peripheral immune system are regulated mainly by Fas/FasL signaling [47, 48]. Activation-induced cell death (AICD) is involved in the elimination of activated T cells and is mainly mediated by Fas–FasL interactions [49, 50]. The results of this study show that there is overexpression of Fas and FasL mRNA and protein in the SAP group, especially in those mice with infectious complications. These results indicate that abnormal expression of Fas and FasL may be major factors involved in the dysregulation of functional lymphocytes.

A significant positive relationship between Fas and FasL protein expression and AI and significant negative relationships between Fas and FasL protein expression and CD4^+^ and CD19^+^ lymphocyte subsets as well as CD4^+^/CD8^+^ ratio were found in the SAP group. Exaggerated inflammatory responses leading to massive release of various cytokines following an excessive activation of lymphocytes and excessive immune response can result in a negative regulation *via* overexpression of Fas and FasL. Subsequent excessive apoptosis of lymphocytes and reduction of lymphocyte subsets can render the host to suffer from the suppression of cellular and humoral immune responses [51, 52]. Our previous study also confirmed that the overexpression of Fas is associated with immunosuppression and sepsis complications in patients with SAP [53].

In conclusion, our study demonstrates that there is an overexpression of Fas and FasL mRNA and protein, excessive apoptosis of lymphocytes, and a marked reduction of circulating lymphocyte subsets in experimental SAP in mice. The results indicate that the overexpression of Fas and FasL is associated with infectious complications and severity of experimental SAP by promoting apoptosis of lymphocytes.

## References

[1] Beger HG (1986). Bacterial contamination of pancreatic necrosis. A prospective clinical study. Gastroenterology.

[2] Renner IG (1985). Death due to acute pancreatitis. A retrospective analysis of 405 autopsy cases. Digestive Diseases and Sciences.

[3] Norman J (1998). The role of cytokines in the pathogenesis of acute pancreatitis. American Journal of Surgery.

[4] Ogawa M (1998). Acute pancreatitis and cytokines: "second attack" by septic complication leads to organ failure. Pancreas.

[5] Ueda T (2006). Immunosuppression in patients with severe acute pancreatitis. Journal of Gastroenterology.

[6] Li JP (2013). Immunosuppression and the infection in patients with early SAP. Front Biosci (Landmark Ed).

[7] Bone RC (1996). Sir Isaac Newton, sepsis, SIRS, and CARS. Critical Care Medicine.

[8] Mentula P (2004). Plasma anti-inflammatory cytokines and monocyte human leucocyte antigen-DR expression in patients with acute pancreatitis. Scandinavian Journal of Gastroenterology.

[9] Ward NS (2008). The compensatory anti-inflammatory response syndrome (CARS) in critically ill patients. Clinics in Chest Medicine.

[10] Kylanpaa ML (2010). Inflammation and immunosuppression in severe acute pancreatitis. World Journal of Gastroenterology: WJG.

[11] Hotchkiss RS (1999). Apoptotic cell death in patients with sepsis, shock, and multiple organ dysfunction. Critical Care Medicine.

[12] Hotchkiss RS (1999). Prevention of lymphocyte cell death in sepsis improves survival in mice. Proceedings of the National Academy of Sciences of the United States of America.

[13] Hotchkiss RS (2000). Caspase inhibitors improve survival in sepsis: a critical role of the lymphocyte. Nature Immunology.

[14] Nagata S, Golstein P (1995). The Fas death factor. Science.

[15] Zhang N (2005). The role of apoptosis in the development and function of T lymphocytes. Cell Research.

[16] da Fonseca RR (2010). Positive selection on apoptosis related genes. FEBS Letters.

[17] Van Parijs L, Abbas AK (1996). Role of Fas-mediated cell death in the regulation of immune responses. Current Opinion in Immunology.

[18] Rieux-Laucat F (1995). Mutations in Fas associated with human lymphoproliferative syndrome and autoimmunity. Science.

[19] Watanabe-Fukunaga R (1992). Lymphoproliferation disorder in mice explained by defects in Fas antigen that mediates apoptosis. Nature.

[20] Ayala A (1999). Increased inducible apoptosis in CD4+ T lymphocytes during polymicrobial sepsis is mediated by Fas ligand and not endotoxin. Immunology.

[21] Dang SC (2008). Dynamic changes of IL-2/IL-10, sFas and expression of Fas in intestinal mucosa in rats with acute necrotizing pancreatitis. World Journal of Gastroenterology: WJG.

[22] Li ZD (2009). Effect of resveratrol-induced FasL up-regulation on the apoptosis of pancreatic acinar cells in rats with severe acute pancreatitis. Nan fang yi ke da xue xue bao = Journal of Southern Medical University.

[23] Gallagher SF (2004). Acute pancreatitis induces FasL gene expression and apoptosis in the liver. The Journal of Surgical Research.

[24] Yasuda T (2002). Splenic atrophy in experimental severe acute pancreatitis. Pancreas.

[25] Hartwig W (2006). Interaction of complement and leukocytes in severe acute pancreatitis: potential for therapeutic intervention. American Journal of Physiology. Gastrointestinal and Liver Physiology.

[26] Alsfasser G (2006). Decreased inflammation and improved survival with recombinant human activated protein C treatment in experimental acute pancreatitis. Archives of Surgery.

[27] Ding SP (2003). A mouse model of severe acute pancreatitis induced with caerulein and lipopolysaccharide. World Journal of Gastroenterology : WJG.

[28] Pastor CM (2004). Role of Toll-like receptor 4 on pancreatic and pulmonary injury in a mice model of acute pancreatitis associated with endotoxemia. Critical Care Medicine.

[29] Chao KC (2006). Blockade of interleukin 6 accelerates acinar cell apoptosis and attenuates experimental acute pancreatitis *in vivo*. The British Journal of Surgery.

[30] Tian R (2013). The role of intestinal mucosa oxidative stress in gut barrier dysfunction of severe acute pancreatitis. European Review for Medical and Pharmacological Sciences.

[31] Schmidt J (1992). Histopathologic correlates of serum amylase activity in acute experimental pancreatitis. Digestive Diseases and Sciences.

[32] Schmittgen TD, Livak KJ (2008). Analyzing real-time PCR data by the comparative C(T) method. Nature Protocols.

[33] Amet N (2009). Insertion of the designed helical linker led to increased expression of tf-based fusion proteins. Pharmaceutical Research.

[34] Ottani A (2009). Vagus nerve mediates the protective effects of melanocortins against cerebral and systemic damage after ischemic stroke. Journal of Cerebral Blood Flow and Metabolism: Official Journal of the International Society of Cerebral Blood Flow and Metabolism.

[35] Zhou YQ (2003). Altered expression of the RON receptor tyrosine kinase in primary human colorectal adenocarcinomas: generation of different splicing RON variants and their oncogenic potential. Oncogene.

[36] Berg RD (1999). Bacterial translocation from the gastrointestinal tract. Advances in Experimental Medicine and Biology.

[37] Wang Q (1994). Increased intestinal marker absorption due to regional permeability changes and decreased intestinal transit during sepsis in the rat. Scandinavian Journal of Gastroenterology.

[38] Moriez R (2005). Myosin light chain kinase is involved in lipopolysaccharide-induced disruption of colonic epithelial barrier and bacterial translocation in rats. The American Journal of Pathology.

[39] Yue C (2012). Lipopolysaccharide-induced bacterial translocation is intestine site-specific and associates with intestinal mucosal inflammation. Inflammation.

[40] Pezzilli R (1995). Circulating lymphocyte subsets in human acute pancreatitis. Pancreas.

[41] Uehara S (2003). Immune function in patients with acute pancreatitis. Journal of Gastroenterology and Hepatology.

[42] Pietruczuk M (2006). Alteration of peripheral blood lymphocyte subsets in acute pancreatitis. World Journal of Gastroenterology: WJG.

[43] Curley PJ (1993). Reduction in circulating levels of CD4-positive lymphocytes in acute pancreatitis: relationship to endotoxin, interleukin 6 and disease severity. The British Journal of Surgery.

[44] Takeyama Y (2000). Peripheral lymphocyte reduction in severe acute pancreatitis is caused by apoptotic cell death. Journal of Gastrointestinal Surgery: Official Journal of the Society for Surgery of the Alimentary Tract.

[45] Hotchkiss RS (1997). Apoptosis in lymphoid and parenchymal cells during sepsis: findings in normal and T- and B-cell-deficient mice. Critical Care Medicine.

[46] Takeyama Y (1998). Thymic atrophy caused by thymocyte apoptosis in experimental severe acute pancreatitis. The Journal of Surgical Research.

[47] Krammer PH (2000). CD95’s deadly mission in the immune system. Nature.

[48] Strasser A (2009). The many roles of FAS receptor signaling in the immune system. Immunity.

[49] Alderson MR (1995). Fas ligand mediates activation-induced cell death in human T lymphocytes. The Journal of Experimental Medicine.

[50] Maher S (2002). Activation-induced cell death: the controversial role of Fas and Fas ligand in immune privilege and tumour counterattack. Immunology and Cell Biology.

[51] Kim K (2002). Nickel(II)-induced apoptosis in murine T cell hybridoma cells is associated with increased fas ligand expression. Toxicology and Applied Pharmacology.

[52] Ayala A (2003). Fas-ligand mediated apoptosis in severe sepsis and shock. Scandinavian Journal of Infectious Diseases.

[53] Qin Y (2013). The role of fas expression on the occurrence of immunosuppression in severe acute pancreatitis. Digestive Diseases and Sciences.

